# A disease-causing variant of COL4A5 in a Chinese family with Alport syndrome: a case series

**DOI:** 10.1186/s12882-021-02585-7

**Published:** 2021-11-13

**Authors:** Jing Wu, Jun Zhang, Li Liu, Bo Zhang, Tomohiko Yamamura, Kandai Nozu, Masafumi Matsuo, Jinghong Zhao

**Affiliations:** 1 Department of Nephrology, the key Laboratory for the Prevention and Treatment of Chronic Kidney Disease of Chongqing, Kidney Center of PLA, Xinqiao Hospital, Army Medical University (Third Military Medical University), Chongqing, 400037 China; 2 Department of Pediatrics, Kobe University Graduate School of Medicine, 7-5-1 Kusunoki-cho, Chuo, Kobe, Hyogo 650-0017 Japan; 3 Department of Physical Therapy, Faculty of Rehabilitation, Kobe Gakuin University, 518, Arise, Ikawadani-cho, Nishi, Kobe, Hyogo 651-2180 Japan

**Keywords:** Alport syndrome, COL4A5, Whole exome sequencing, Splicing error, Minegene assay

## Abstract

**Background:**

Alport syndrome (AS), which is a rare hereditary disease caused by mutations of genes including COL4A3, COL4A4 and COL4A5, has a wide spectrum of phenotypes. Most disease-causing variants of AS are located in the exons or the conservative splicing sites of these genes, while little is known about the intronic disease-causing variants.

**Methods:**

A Chinese AS family was recruited in this study. All the clinical data of AS patient were collected from medical records. After pedigree analysis, the pathogenic variants were studied by the whole exome sequencing (WES). Minigene assay and in vivo RT-PCR analysis were performed to validate the functions of the variants.

**Results:**

Renal biopsy showed a typical histopathology changes of AS. WES revealed compound heterozygous substitution, NM_033380 c.991–14(IVS17) A > G, in the intron 17 of the COL4A5 gene, which were confirmed by Sanger sequencing. Moreover, the variant was co-segregated with the phenotype in this family. Minigene assay in cultured cell lines showed that a splicing error was induced by this intronic variant, which further confirmed by in vivo RT-PCR analysis.

**Conclusion:**

A novel intronic disease-causing variant in COL4A5 gene was identified by WES, which was the molecular pathogenic basis of AS.

## Background

Alport syndrome (AS) is a hereditary nephropathy, whose phenotypes ranged from isolated hematuria with a non-progressive course to progressive renal disease with extrarenal abnormalities [1–3]. The molecular basis of AS is related to the mutant genes including COL4A3, COL4A4 and COL4A5.

The clinical diagnose of AS is mainly based on clinical manifestations and renal histopathology. When the clinical manifestations are atypical, genetic testing is powerful to establish an accurate diagnosis of AS. Due to the large sizes of these genes and the absence of mutation hot spots, PCR-based screening of the variants of AS patients is much complicated and time-consuming [4, 5]. With the progress in next-generation sequencing (NGS), a strategy by utilizing targeted capture to analyze COL4A3, COL4A4, and COL4A5 is much powerful [6]. However, with the expense of whole exon sequencing (WES) goes down, WES has been extensively applied in clinical practice [7]. For example, WES was successfully utilized to identify de novo mutations in COL4A5 in two Korea girls with AS [8]. Moreover, WES might be a better choice when the clinical manifestations are atypical.

Besides the coding regions, WES can also detect the adjacent intronic variants. Although genetic analysis of inherited diseases is important, it is still difficult to distinguish intronic variants leading to splicing errors from harmless polymorphisms. Several in silico approaches have been developed to assess the function of sequence variants, but the fundamental method to analyze splicing errors is by in vivo assay [9, 10]. Recently, a hybrid minigene assay has been developed to analyze the function of intronic variants associated with splicing errors [11]. For the pathogenic variants of AS, most variants are located at exons and the conservative splicing sites, while the functional intronic variants are very limited. In current study, we reported a functional intronic variant in a Chinese family. a functional splicing assay using.

## Methods

### Subjects

The study was approved by the Ethics Committee of Xinqiao Hospital at Army Medical University (Chongqing, China). All participants provided written informed consent. The proband was a 9-year-old Chinese Han girl. Her family including her parents and her grandparents was recruited in current study. Blood samples were collected for DNA isolation.

### Clinical evaluation

Clinical data was obtained from electronic health records. Abdominal ultrasound examination and histopathology study of renal biopsy were performed for clinical diagnosis.

### DNA extraction

Genomic DNA was isolated from the peripheral blood cells of the pedigree with a QIAamp DNA Blood MiniKit (Qiagen, Germany), according to the manufacturer’s instructions.

### Exome sequencing

Whole exome sequencing of the DNA samples from the proband and the parents was performed by Chigene (Beijing) Translational Medical Research Center (Beijing, China), as previously described [12].

### In silico splicing assay

A splicing effect of detected mutation was predicted via the Alamut Visual v.2.11 software (Interactive Biosoftware, Rouen, France) by using following algorithms; SpliceSiteFinder-like, MaxEntScan, NNSPLICE, and GeneSplicer.

### Hybrid minigene assay

The DNA fragment spanning the exon 17 ~ 19 of Col4A5 was obtained by PCR, and was cloned into the minigene vector H492 [11]. Then the recombinant vector was transfected into HEK293T and Hela cells. Twenty-four hours later, total RNA was isolated and RT-PCR was performed to analyse the splicing of recombinant vector, and the product was confirmed by Sanger sequencing.

### In vivo assay

Subcutaneous adipose tissue was obtained from the patient, and total RNA was isolated. RT-PCR was performed to amply a cDNA fragment spanning the exon 16 ~ 20 of Col4A5. Briefly, total RNA was extracted by using an RNA Isolation Kit (TakaRa, Dalian, China) and was reverse-transcribed into cDNA. Then regular PCR was performed on a cycler with Col4A5 primers: 5′- AAAGAGGTAAACCAGGCAAAGA-3′ and 5′- ATCACTAGGAGGAATGTGAGGG-3′. The product of RT-PCR was confirmed by Sanger sequencing.

## Results

### Clinical presentations

A 9-year-old Chinese Han girl was admitted to our Department for microscopic hematuria. Five year ago, she suffered from proteinuria and urine occult blood (3+), and took some renoprotective drugs to ameliorate proteinuria. However, urine occult blood was persistent positive. After that, no additional treatment was executed, as no other symptoms were found.

**Fig. 1:**
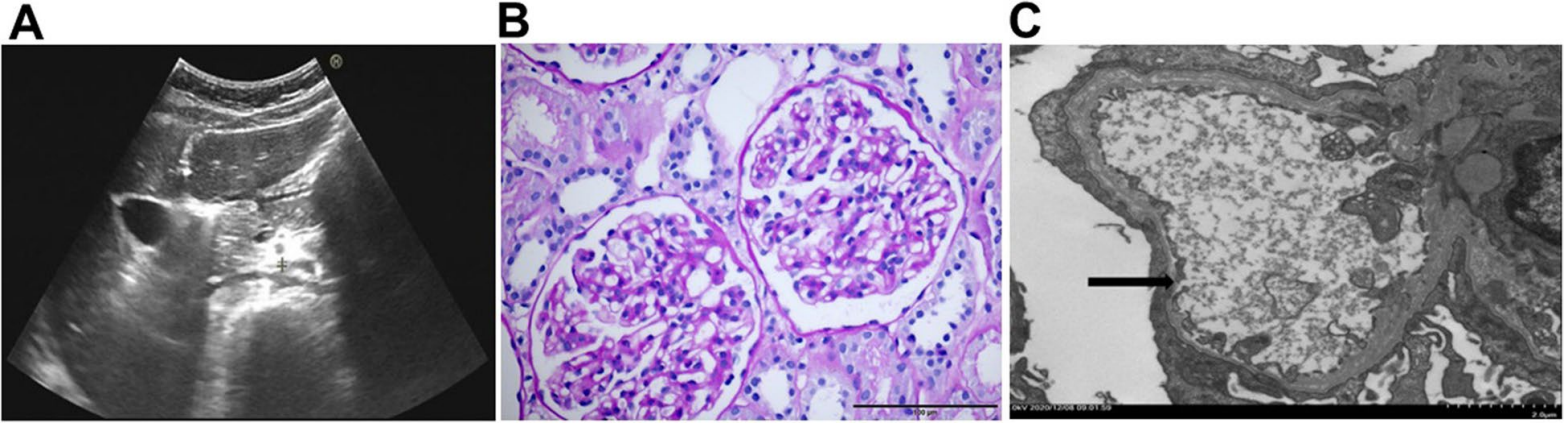
Clinical diagnostic images of the proband. **A** Abdominal ultrasound image. **B** Histopathology study of renal biopsy. **C** Electron microscopic examination of renal biopsy. Bar, 100 μm. Bold arrow indicated the thinning and splitting of GBM. Bar, 2.0 μm

Urine routine test showed that urine protein (3+) and urinary occult blood (3+) were abnormal. But no other parameters were revealed by other laboratory tests including blood routine tests, serum chemistry and immunology. Pure tone audiometry result was unremarkable. No obvious abnormality was detected by abdominal ultrasound examination and electrocardiographic examination except compression and dilation of left renal vein (Fig. 1a).

Then, histopathology study of renal biopsy from the proband was performed to further understand its renal pathology. Totally, 12 glomeruli were observed, with one glomerulus being ischemic sclerosis. Mesangial cells and mesangial stromal segments were mildly proliferated, and renal tubules were filtered with foam cells (Fig. 1b). All the immunological staining including IgA, IgG, IgM, complement C3, C4, C1q, K and λ was negative. Segmental thinning, irregular thickening and splitting of the glomerular basement membrane (GBM) were observed by electron micrographs (Fig. 1c). The histopathology changes fitted well with that of AS.

The proband’s mother was diagnose as chronic glomerulonephritis when she was 20 years old because of microscopic hematuria. But the symptom was alleviated after residential treatment. She had no conscious symptoms until her daughter was hospitalized. Her urine routine test showed positive urine protein (2+) and urinary occult blood (3+).

According to the renal biopsy and the family history, the proband received a treatment of angiotensin-converting enzyme inhibitor (ACEi, Irbesartan, 80 mg/day), as well as traditional Chinese medical to protect renal function. Follow-up data showed that the UPCR ratio (urinary protein/creatinine) decreased from 2355.5 mg/g to 1154.5 mg/g about 1 month later, 1144.8 mg/g at 3 month later, and 308.6 mg/g about 1 year later.

### Genetic analysis

**Fig. 2:**
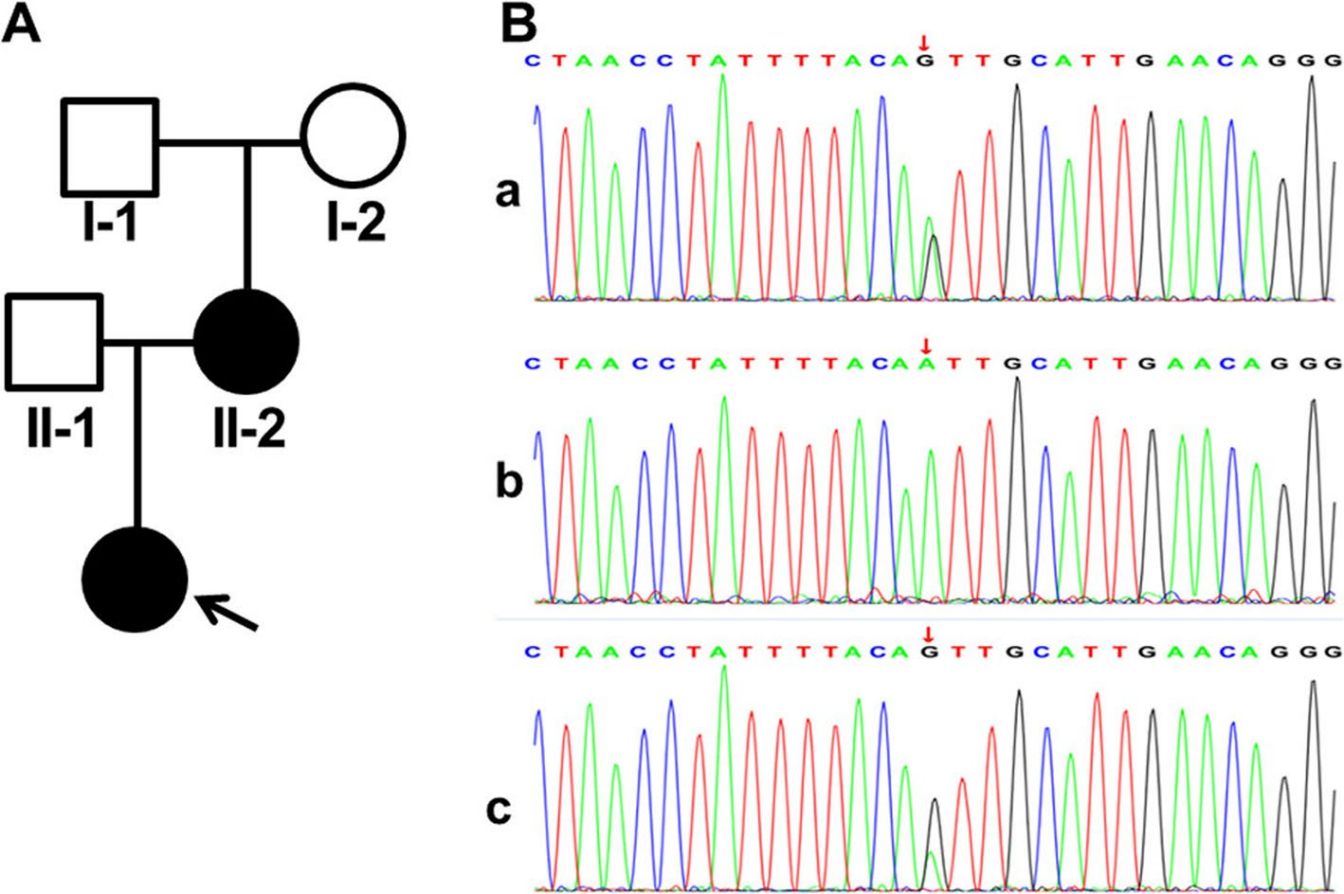
Genetic analysis of the Chinese family. **A** Pedigree of the Chinese family. Affected family members are denoted in black. Arrow indicates the proband; **B** Direct Sanger sequencing confirmed the heterozygous mutations of COL4A3 gene. a, proband; b, II-1; c, II-2

After investigating the family history, diagnose of AS was highly suspected in this family (Fig. 2a). To make a conclusive diagnosis, the proband and her parents were recommended to have a genetic test. After sequencing, a heterozygous substitution, NM_033380 c.991–14(IVS17) A > G, was revealed. Both the mother and the proband were heterozygous, but her grandparents were wild genotype. The variant was further confirmed by Sanger sequencing (Fig. 2b). This variant was excluded from the Single Nucleotide Polymorphism database (dbSNP) and the ClinVar database. In addition, the variant can be classified as “Uncertain significance” according to the American College of Medical Genetics and Genomics (ACMG) standards and guidelines [13].

### Mutation analysis

**Fig. 3:**
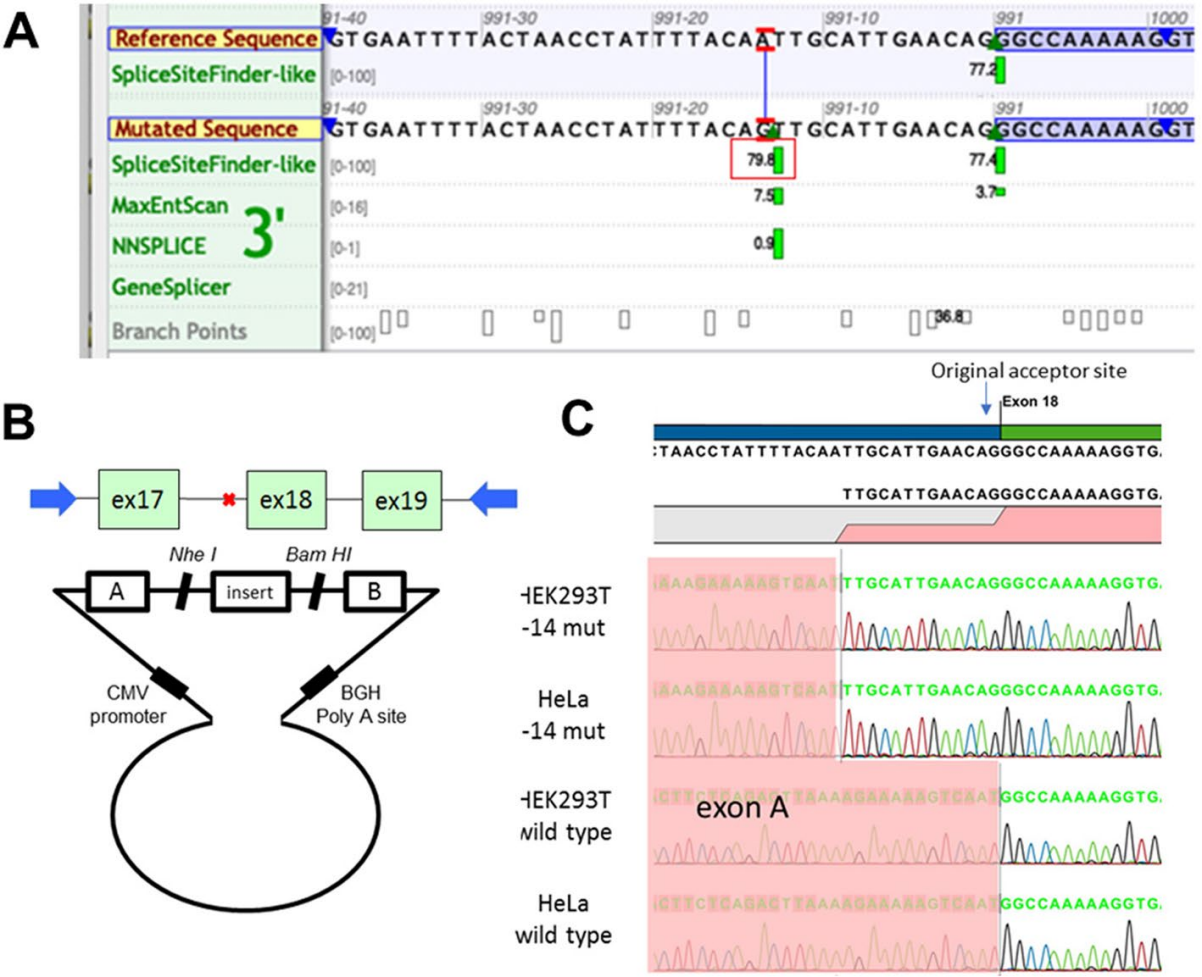
Mutation analysis of the intronic variant. **A** Analysis using Alamut Visual v.2.11 has shown that the variant c.911-14A > C generated the novel potential splicing acceptor site (red rectangle). **B** Exon 17 to 19 spanning the intronic variant was cloned into vector H492. **C** Splicing products in cell lines were confirmed by Sanger sequencing

As the variant located in the intron 17, we suspected it might affect the splicing. First, we analyzed the influence of the variant on splicing using the Alamut Visual v.2.11 software (Interactive Biosoftware, Rouen, France). As shown in Fig. 3a, an additional splicing acceptor site was generated by the variant, which was a little stronger than the original one. Therefore, the DNA fragment spanning the exon 17 ~ 19 of Col4A5 was amplified and cloned into minigene vector (Fig. 3b). After transfect, the spliced RNA from the hybrid minigene had additional 13-bp fragment, which was confirmed by Sanger sequencing (Fig. 3c). Therefore, this intronic variant is functional.

### In vivo validation

**Fig. 4:**
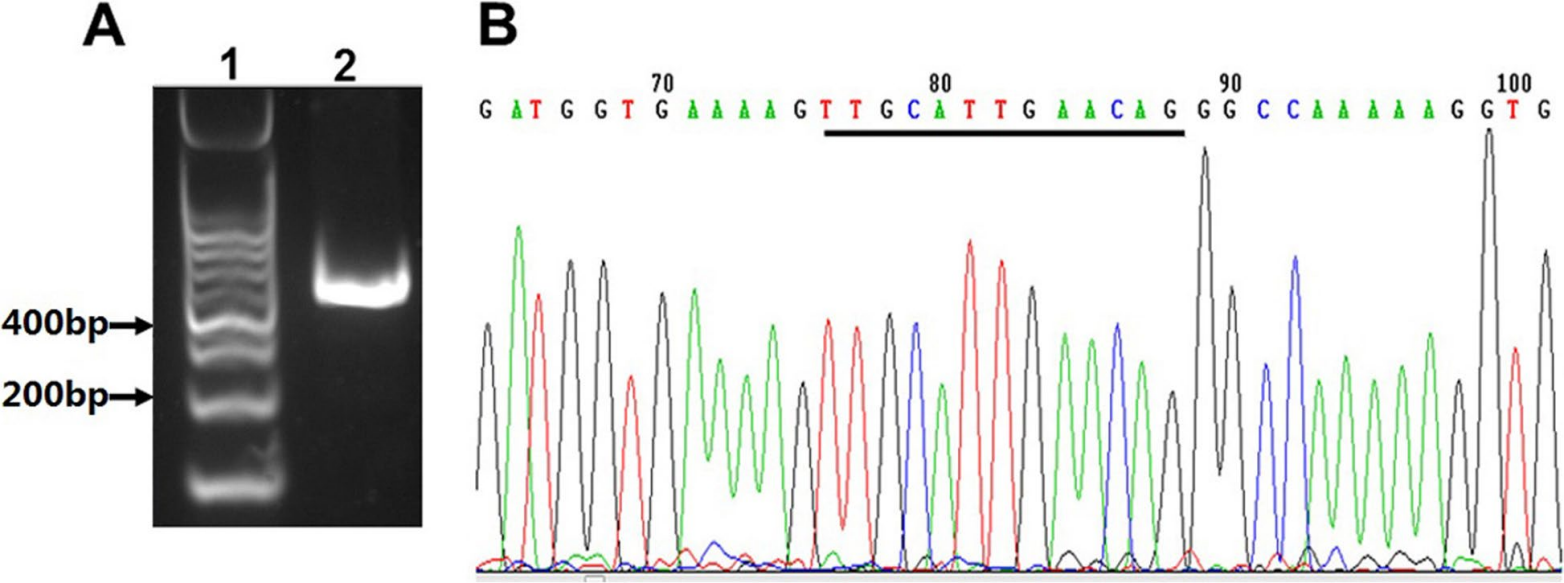
In vivo validation of the splicing of COL4A5 gene. **A** Total RNA was obtained from the fat tissue, and was analyzed by RT-PCR. 1, DNA ladder; 2, RT-PCR product. **B** RT-PCR product was directly sequenced. Black line indicated the additional fragment

To confirm the result of minigene assay, we further analyzed the splicing aberration in the patient, which might be the most reliable. For this purpose, subcutaneous adipose tissue was obtained from the patient, and total RNA was isolated. As shown in Fig. 4a, a corresponding DNA band was detected. Subsequently, Sanger sequencing revealed that an additional fragment of 13-bp was confirmed, which would lead to reading frame shift (Fig. 4b).

## Discussions

In current study, we reported a disease-causing variant in intron of COL4A5 gene. The pathogenic mutation in COL4A5 gene was identified by WES and confirmed by subsequent Sanger sequencing and functional analysis.

AS is a rare genetic disorder that caused by pathogenic variants in COL4A3, COL4A4, and COL4A5 that result in abnormalities of the collagen IV α345 network of basement membranes. Its phenotypes are complicated, which can vary from isolated hematuria with a non-progressive to progressive renal disease with extrarenal abnormalities. Most AS cases will deteriorate to ESRD within the first three decades of their lives [14]. For the AS cases, microhematuria is the most frequently observed symptom, although some individuals are asymptomatic. A proportion of patients eventually develop proteinuria. Both the proband and her mother were found to have microhematuria and proteinuria. However, the symptoms of the mother were a little lighter than that of the proband.

Variants in COL4A5 count for about 80–85% of AS patients [15]. To date, there are more than 1300 variants of COL4A5 gene deposited in the ClinVar database. More and more variants are discovered by WES technology [16]. All of these variants include deletion, duplication, substitution and splicing mutation. Among these variants of small indels, most variants are pathogenic or likely pathogenic. In previous reports regarding the splicing mutation, the variants are almost located at the evolutional conserved sites within the boundary of exons and introns. Few reports focused on the functions of intronic variants, becuase it is hard to distinguish intronic variants leading to splicing errors from harmless polymorphisms. Recently, Chiereghin C et al. reported an intronic disease-causing variant in COL4A5. Their variant (c.2245-40A > G) was outside the conventionally screened candidate region for genetic diagnosis, but was functional by using a minigene-based approach in HEK293 cells [17]. According to the ACMG guidelines, our variant is classified as Uncertain Significance. However, based on its co-segregation with the phenotype in this family, and its influence on the splicing, this variant is finally classified as pathogenic in this study.

RNA splicing is more complicated than expected. Besides the conservative splicing donor and acceptor site, additional sequences known as splicing enhancers and silencers can also facilitate exon selection by the spliceosome [18, 19]. These enhancers and silencers can be located at exons or introns, acting as binding sites for splicing factors like the serine/arginine-rich (SR) protein. In turn, variants involved in these elements also lead to splicing errors. For example, exon skipping was induced by a nonsense mutation in the DMD gene, which led to the conversion of a splicing enhancer to a splicing silencer [20]. Therefore, besides splicing donor or receptor sites, other intronic variants need to be carefully analyzed.

Currently, one of the most efficient reproaches to functionally analyze the intronic variants is minigene-based approach, when the in vivo splicing study cannot be available. Horinouchi T et al. reported a hybrid minigene system to analyze the intronic variants and their results showed 6 of seven tested intronic variants were functional [11]. By using this method, we further confirm the splicing error caused by this variant. As COL4A5 gene locates on X-chromosome, only one type of the splicing product was detected by RT-PCR analysis in this study, which might be due to random X inactivation [21, 22]. This might also be the reason why female X-linked AS patients have much complicated clinical presentations. Nevertheless, the main limitation of current study is that the convincing of intronic pathogenic variant is time-consuming. The genetic information cannot benefit in-patient treatment timely, but for long-term follow-up and genetic counseling.

## Conclusion

In summary, the pathogenic intronic variant in COL4A5 was identified by WES in a Chinese AS family and its effect on splicing was verified by minigene assay and in vivo study. Identification of the pathogenic variant helps to understand the relationship between phenotypes and genotypes of AS.

## Data Availability

The original data of WES are not available according to the Chinese policies of Human Genetic Resource, but the VCF file is available from the corresponding author on reasonable request. Data Review link: https://db.cngb.org/cnsa/review/show/CNP0001544_20210201_0c2973d5/

## Abbreviations

AS: Alport syndrome
ACMG: American College of Medical Genetics and Genomics
ESRD: End-stage renal disease
GBM: Glomerular basement membrane
NGS: Next-generation sequencing
SNVs: Single nucleotide variants
dbSNP: Single Nucleotide Polymorphism database
WES: Whole exome sequencing
